# Epigallocatechin-3-gallate preconditioned Adipose-derived Stem Cells confer Neuroprotection in aging rat brain

**DOI:** 10.7150/ijms.46696

**Published:** 2020-07-19

**Authors:** Dennis Jine-Yuan Hsieh, Lawrence Marte, Wei-Wen Kuo, Da-Tong Ju, William Shao-Tsu Chen, Chia-Hua Kuo, Cecilia Hsuan Day, B. Mahalakshmi, Po-Hsiang Liao, Chih-Yang Huang

**Affiliations:** 1 School of Medical Laboratory and Biotechnology, Chung Shan Medical University, Taichung, 402, Taiwan.; 2 Clinical Laboratory, Chung Shan Medical University Hospital, Taichung 402, Taiwan.; 3 Department of Healthcare Administration, Asia University, Taichung, 413, Taiwan.; 4 Department of Biological Science and Technology, China Medical University, 404, Taichung, Taiwan.; 5 Department of Neurological Surgery,Tri-Service General Hospital, National Defense Medical Center, Taipei, Taiwan.; 6 Department of Psychiatry, Hualien Tzu Chi Hospital, Buddhist Tzu Chi Medical Foundation, Hualien 970, Taiwan.; 7 School of Medicine Tzu Chi University, Hualien 970, Taiwan.; 8 Department of Sports Sciences, University of Taipei, Taipei, 111, Taiwan.; 9 Department of Nursing, MeiHo University, Pingtung, 912, Taiwan.; 10 Institute of Research and Development, Duy Tan University, Da Nang 550000, Vietnam.; 11 Division of General Surgery, Department of Surgery, Shuang Ho Hospital, Taipei Medical University, New Taipei, 235, Taiwan.; 12 Department of Medical Research, China Medical University Hospital, China Medical University, Taichung, 404, Taiwan.; 13 Department of Biological Science and Technology, Asia University, Taichung 413, Taiwan.; 14 Cardiovascular and Mitochondria related diseases research center, Hualien Tzu Chi Hospital, Buddhist Tzu Chi Medical Foundation, Hualien 970, Taiwan.; 15 Center of General Education, Buddhist Tzu Chi Medical Foundation, Tzu Chi University of Science and Technology, Hualien 970, Taiwan.

**Keywords:** Adipose-derived mesenchymal stem cells, epigallocatechin gallate, aging, neuroprotection

## Abstract

Aging is the most important current issue and is usually accompanied by complications, such as cardiovascular disorders and neurodegenerative diseases, which are the leading causes of death worldwide and the second major cause of death in Taiwan. In this study, we have investigated the protective effect of adipose-derived mesenchymal stem cells (ADSCs) and the role of epigallocatechin gallate (EGCG) in enhancing this effect in aging cerebral cortex of rats. Further, we attempted to elucidate the molecular mechanism through which EGCG influences the protective effects of ADSC. ADSCs, co-cultured with EGCG, were injected into 20-month-old Wistar rats. Hematoxylin and eosin staining of the cerebral cortex revealed noticeable neurogenic activity and visible improvements in the integrity of the pre-frontal cortex tissue, compared to that in rats treated with ADSCs alone. Western blot analysis confirmed that ADSC, co-cultured with EGCG, enhanced cell survival via the p-Akt pathway and improved mitochondrial biogenesis via the SIRT-1 pathway. Moreover, it increased the available brain-derived neurotrophic factor to a higher degree than that in the ADSC group. Furthermore, western blotting showed that EGCG improved the antioxidant activity of the ADSCs in the cortex tissues via the Nrf-2 and HO-1 pathway. Based on these findings, we propose that this variation in stem cell treatment may facilitate functional recovery and enhanced neuroprotection in aged brains.

## Introduction

Aging is one of mankind's most aggressive battles. Until modern times, humans had to focus on developing cures and vaccines for ever-present immediate dangers and diseases of environmental origin, which resulted in mortality at an early age. Development in medical technology and sanitary conditions has resulted in longer fuller lives and has increased our understanding of the human body. Living longer has always been the goal, and as mankind slowly succeeded in managing illnesses with external origins, our focus turned inwards on disorders with internal origin that commonly affect the aged. Aging was only more formally classified as a conquerable disease in 1983 1, 2-4. A 2013 review drew parallels between cancer and aging, and suggested that, although seemingly different, each “can be regarded as two different manifestations of the same underlying process—namely, the accumulation of cellular damage” 5. Cumulative cellular and molecular damage leading to a loss of function is, in essence, the definition of aging. As an organism ages, DNA damages accumulate and stem cell reserves are unable to maintain proper tissue homeostasis and function, resulting in the loss of self-healing ability, which the organism attempts to compensate by up-regulating the pathways involved; however, these pathways that originally served a useful purpose, exacerbate the problem and cause irreversible damage under excessive conditions, as observed in the case of reactive oxygen species (ROS) stress 6. Moreover, another study re-evaluated the role of ROS during aging, and reported that it normally induces cell propagation via a cell signal 7, and knock-out organisms, with higher ROS production, tended to live longer 8.

The cerebral cortex is the most evolved structure in the mammalian brain, with a prominent role in governing cognition, behavior, emotion, and decision-making 9. However, this region is highly impacted by aging and reportedly begins to atrophy and thin to the point where magnetic resonance imaging shows a noticeable difference by middle age 10. The aging cortex may cause the development of unusual behavior and decision-making ability, which is modulated in humans by familial and social buffers that help to prevent grave errors in judgement. However, in case of aged animals in the wild, unnecessary risks combined with impaired motor function, can be fatal. Therefore, one of the organs most affected by aging is the brain, particularly the cerebral cortex region.

Adipose-derived stem cells (ADSC) reportedly provide neuroprotection if administered intravenously to ischemic stroke rat models shortly after inducing a stroke, and induce neurogenesis and repair when administered several weeks after the stroke 11. This indicates the ability of ADSCs to adequately permeate the blood brain barrier and provide relief to the damaged area. ADSCs are pluripotent and are thus able to differentiate themselves depending on their surrounding tissue. Thus, such holistic approach to therapy would be beneficial for patients with stroke, who are generally at an advanced age and weakened state at stroke onset. Furthermore, damaged neural tissues evidently respond positively to the ADSC extract, even in tissues where donor cells are not found, confirming that secreted factors likely confer the therapeutic effects, which may help integrate both host and donor cells to repair brain damage more robustly 12. Epigallocatechin-3-gallate (EGCG) is an antioxidant and bioactive polyphenol found in green tea (*Camellia sinensis*), which is consumed around the world. Previous experiments have confirmed that it is capable of not only permeating the blood brain barrier but also inducing nerve cell proliferation in rat cerebral cortices, as well as potentially counteracting axonal growth inhibitors and creating a local environment conducive to neural growth 13-15. EGCG was shown to positively affect highly damaged post-ischemic stroke cortex tissue by attenuating ROS and up-regulating glutathione and NRF-2 along with the antioxidant response element pathway, which eventually decreases the infarction size 16. Normally, EGCG increases the mitochondrial membrane potential and oxidative phosphorylation in both neurons and astrocytes by activating cytochrome C oxidase, which causes a net increase in the production of ATP and, at higher doses, ROS 17. In *C. Elegans*, EGCG was reported to increase cellular metabolism in tandem with ROS production, which subsequently activated antioxidant and cellular proliferation response 18. This increased the longevity and rate of the mitochondrial biogenesis of the organism in a dose dependent manner via the activation of AMP-activated protein kinase (AMPK) and Sirtuin-1 (SIRT-1). However, this effect was the strongest in the first half of the organism's life and tapered off afterwards.

SIRT-1, a well-known histone deacetylase, is considered to be partly responsible for the enhanced lifespan of experimental models subjected to dietary restrictions. However, it also deacetylates p53, a powerful tumor suppressor and its first known nonhistone target, with which it exhibits a strong interplay in tumorigenesis and senescence 19. p53 is also known to be activated under conditions of increased ROS or aging as well as by chemical means that result in these factors 20. While the role of SIRT-1 in aging remains controversial, recent studies have made significant progress in elucidating how SIRT-1 overexpression in the brain, specifically the dorsomedial and lateral hypothalamic nuclei, can increase the lifespan of mice when combined with increased expression of orexin type 2 receptor 21. Thus, the functional complexities of this key protein and its role with reference to p53 remain to be elucidated.

Aging and even a poor diet can decrease an organism's antioxidant defenses in a pathological manner 22. Heme oxygenase 1 (HO-1) is an enzyme that catalyzes the conversion of pro-oxidant free-floating heme into ferritin and bilirubin, both of which have antioxidant properties. A byproduct of this conversion is carbon monoxide, which, in appropriate quantities, ultimately induces anti-apoptotic and anti-inflammatory factors and may even activate nuclear factor erythroid 2-related factor 2 (Nrf-2), which is an upstream signaling protein for HO-1 23. EGCG is a potent activator of Nrf-2, and recent studies have shown that it functions by disabling the Nrf-2 inhibitor, Kelch-like ECH-associated protein 1 (Keap1), which normally keeps Nrf-2 bound to itself in the cell cytoplasm 24. Thus, activation of both these proteins results in an increased antioxidant capacity, as the antioxidant response element within the cell is also activated, thereby protecting the cell from oxide stress induced death.

EGCG was found to provide beneficial effects in adult hippocampal region by regulating Akt and ERK phosphorylation 25. ADSCs could differentiate to neuron-like cells and present neuronal properties 26. Therefore, in this study, we investigated the combination protective effect of ADSCs and EGCG in aging cerebral cortex of rats. Moreover, we attempted to elucidate the molecular mechanism through which EGCG influences ADSC protective effects in this aging rat model, particularly focusing on Sirt-1, PGC-1α and p-AMPKα, that are the proteins involved in the molecular pathways that promote longevity, as well as brain-derived neurotrophic factor (BDNF).

## Materials and Methods

### Animal model and ADSC experimental procedure

Twenty-eight male Wistar rat pups (*Rattus norvegicus*; 8 weeks old) were purchased from LASCO Biotechnology (Taipei, Taiwan). The animals were maintained in plastic cages with sawdust bedding and fed a standard diet, with filtered water access provided *ad libitum*. They followed a 12 h light-dark cycle, with a controlled ambient temperature, and were acclimatized for one month before commencing the experimental procedure. The Institutional Animal Care and Use Committee of China Medical University, Taichung, Taiwan approved all protocols (No. 2017-379-1).

ADSCs were isolated from the adipose tissue of the 3-month-old rats, following a previously described method 27. The primary ADSCs were then cultured in a low-glucose Dulbecco's Modified Eagle's Medium (DMEM-D2902; Sigma-Aldrich, St. Louis, MO, USA) with 10% Fetal Bovine Serum and 1% penicillin/streptomycin. On reaching 80% confluence, the cells were subcultured with a 0.25% trypsin/0.02 mM EDTA solution and seeded at a density of 1 × 10^4^ cells/cm^2^. The rats were divided into five groups: Control group (N=7); ADSC group (N=6); ADSC + EGCG group (N=6) (ADSCs pre-conditioned with 10 µM EGCG for 2 h); ADSC + miR-3575 mimic group (N=4) (ADSCs transfected with 10 nM miR-3575 for 24 h); and ADSC + miR-3575 inhibitor group (N=5) (ADSCs transfected with 20 nM miR-3575 inhibitor for 24 h). Rats in each group were treated with 1 × 10^6^ ADSCs by tail vein injection at 20 months of age and were sacrificed at the age of 22 months for analyses.

### Tissue extraction

Whole brains of all rats were extracted, blood was removed with ice-cold Phosphate-buffered Saline (PBS), and the brain tissues were placed in 4% paraformaldehyde. The cortex tissue was separated from the hippocampus, focusing on the prefrontal cortex. The tissue was homogenized in lysis buffer (iNtRON Pro Prep, cat. 17081; 3 mL/100 mg tissue) and then stored at -80 °C for 12 h. The homogenates were then placed on ice for 15 min and centrifuged at 12,000 rpm for 40 min. The supernatant from each sample was collected and stored at -80 °C for further analyses.

### Hematoxylin-Eosin (H&E) Staining

The cortex tissues were first placed in formalin and then encased in paraffin. The slides were immersed in a series of ethanol concentrations (100%, 95% and 75%) for 15 min each, stained with H&E, and finally rinsed with water. The slides were then dehydrated using serial ethanol concentrations for 15 min each, cleaned with xylene, which was followed by placing coverslips on them. Photomicrographs of the prefrontal cortex, focusing on the same area in all samples, were taken using a Zeiss Axiophot microscope (Zeiss, Oberkochen, Germany) at 200× magnification to better observe the cell morphology of the tissues.

### Protein Quantification

Protein content in the cortex tissue extract was determined by Bradford protein assay, which was performed using a protein dye (Bio-Rad, Richmond, CA, USA, cat. 500-0006) and bovine serum albumin (BSA; UniRegion Bio-Tech, USA). BSA was used as the protein standard prepared by serial dilutions with double deionized water (ddH2O); it was placed in a 96-well plate to which the protein assay dye was added and incubated at room temperature for 5 min. Changes in the optical density were then measured at 595 nm and plotted to obtain a standard curve. The cortex samples (previously collected supernatant) were diluted with ddH2O and loading dye, as required, and were first placed on a 100 °C-hot plate for 10 min, and then on an ice bath for 10 min before the assay. Next, they were added to the protein dye in a 96 well plate, the absorbance was measured at 595 nm, and protein concentration was determined by comparing the results against the standard curve for each sample. Quantification of the cortex samples was conducted in triplicate.

### Western Blot analysis

SDS-PAGE was performed with 8-13.5% polyacrylamide gels to denature and separate the proteins before transferring them to a 0.45 µm polyvinylidene difluoride membrane (PVDF) (Millipore, USA, IPVH00010) and then blocking the membrane with 5% milk (Anchor, New Zealand) prepared with 100 mM Tris-HCl, 0.9% NaCl, and 0.1% (vol/vol) Tween 20, adjusted to pH 7.4 (TBST), for 1 h at room temperature to prevent non-specific binding. The membranes were then incubated at 4 °C overnight, with the relevant primary antibodies in TBST at a 1:2000 dilution. The membranes were washed in TBST three times for 10 min each, and were then incubated with the appropriate horseradish peroxidase (HRP) conjugated secondary antibodies (Santa Cruz Biotech, sc-2004, sc-2005, sc-2020) for 1 h at 1:4000 dilution. After washing the membranes as described before, they were placed in a Chemiluminescent HRP substrate (Millipore, Darmstadt, Germany, WBKLS0500), and the immunoblotted proteins were quantified using a Fujifilm LAS-4000 imaging system (Fujifilm, Tokyo, Japan). All quantitative analyses were conducted with ImageJ (NIH, MD, USA) and Excel 2016 (Microsoft, USA).

### Statistical analyses

Statistical analysis was performed by one-way analysis of variance (ANOVA) and Student's *t*-test using SigmaPlot 10.0 software (Systat Software Inc., San Jose, CA, USA) with GraphPad Prism 8. For all tests, *P* < 0.05, *P* < 0.01, or *P* < 0.001 were considered statistically significant.

## Results

### ADSC and ADSC + mimic treatments resulted in moderate neuroregeneration, whereas ADSC + EGCG treatment showed a marked improvement in brain tissue

**Figure 1:**
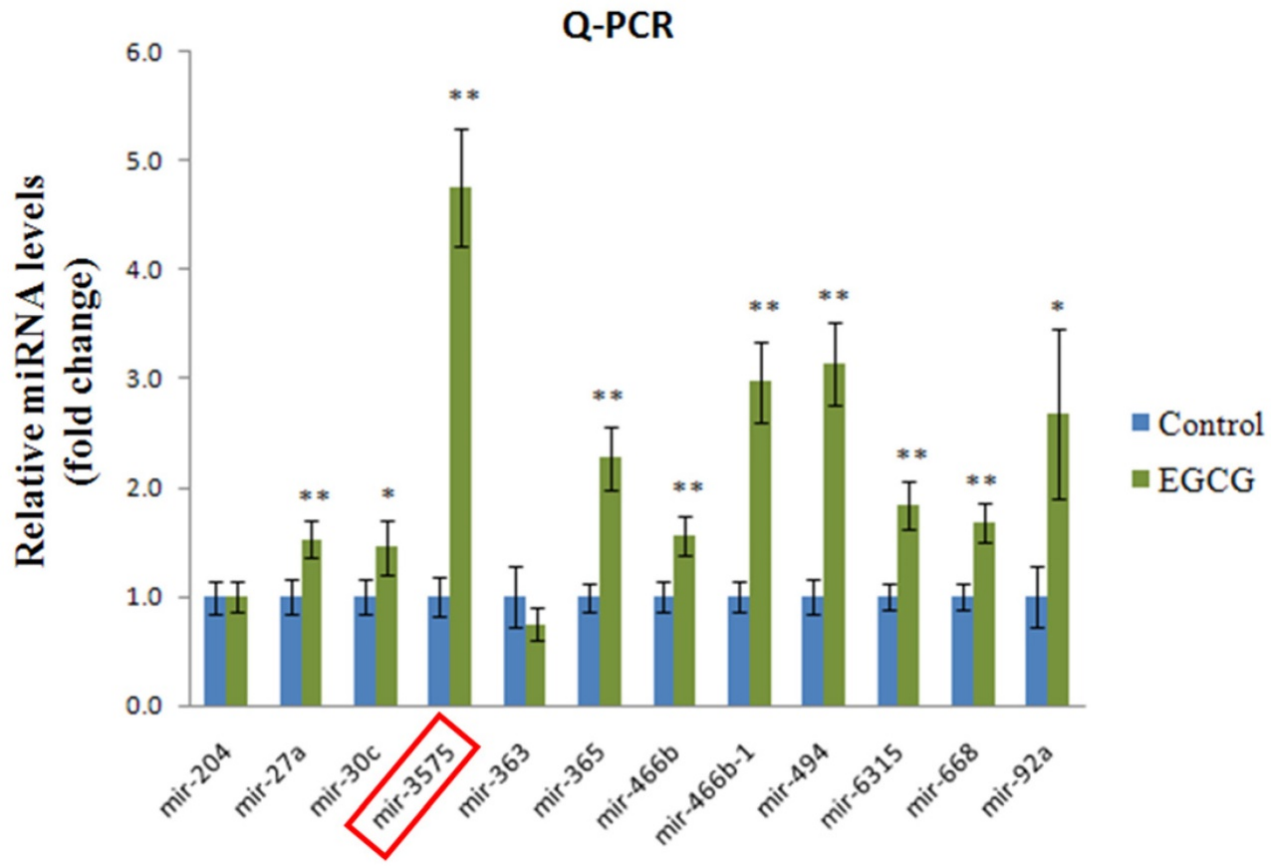
** Expression of miR-3575 in brain tissue section.** Expression of miR-3575 increased in ADSCs after EGCG treatment.

**Figure 2:**
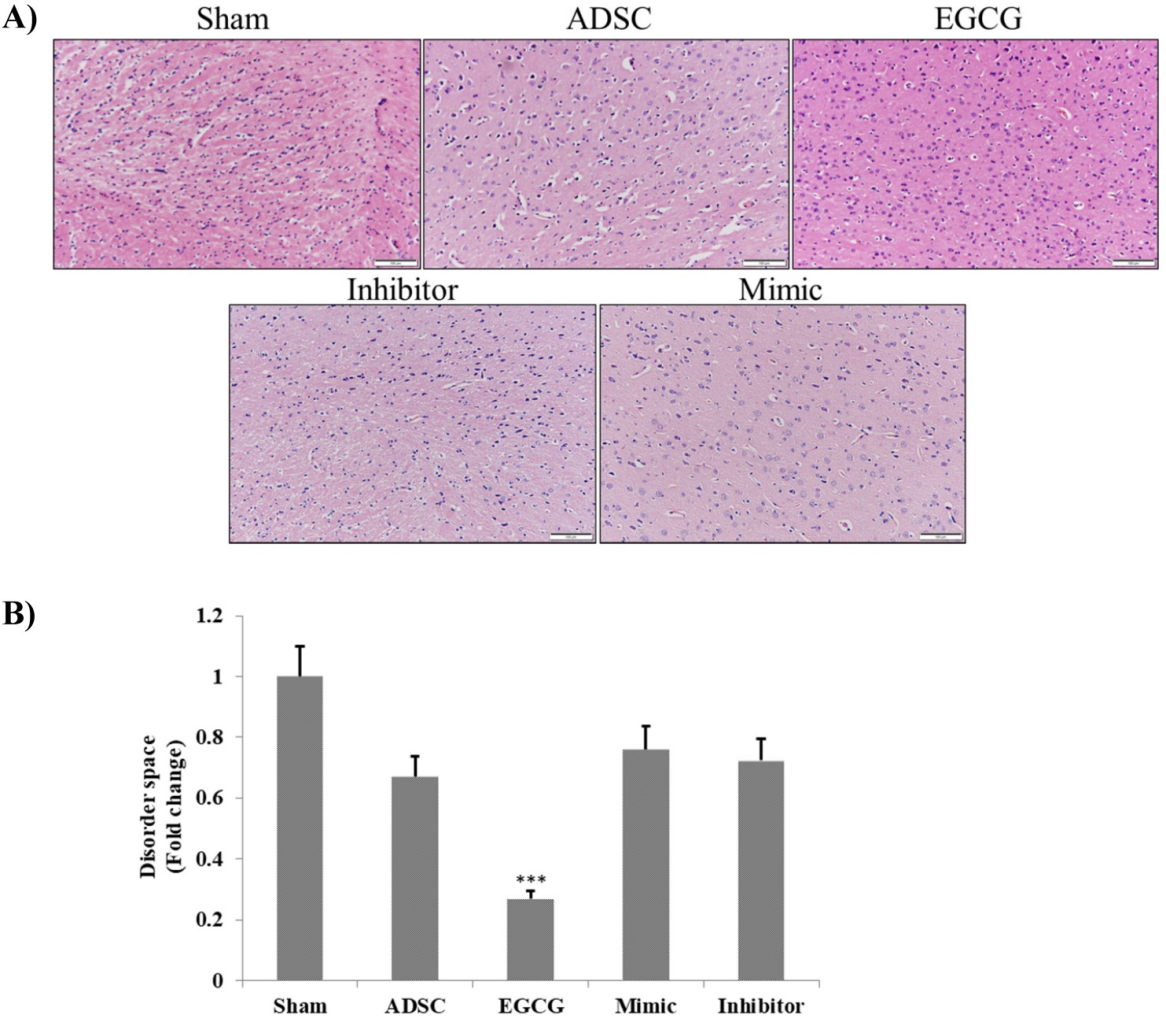
** Brain tissue section H&E staining assay. A.** H&E photomicrographs (200× magnification) show a clear difference between the degenerative lesions of the aged control rats and treated groups. ADSC and ADSC + mimic groups both showed a minor improvement, while the ADSC + EGCG group showed major improvements, with the latter displaying increased cell proliferation. The ADSC + inhibitor group showed the least improvement. Scale bars: 100 µm.** B.** Quantification and verification of the above-mentioned differences between the treatment groups. Data are presented as the mean ± SEM of three separate micrographs. ***, P< 0.001 compared with sham group.

To determine the effect of EGCG on ADSCs, we measured the expression of miR-3575 in untreated ADSCs and those preconditioned with EGCG. We found that EGCG increased the expression of miR-3575 in ADSCs (**Figure 1**). H&E staining revealed distinct differences in the overall integrity of the cortex sections between the groups (**Figure 2A**). In the aged control group, the striations of the tissue disorders space were easily noticeable and linear. ADSC injection improved the integrity of the tissue with many of the smaller pockets filled, and the results were mirrored in the ADSC + mimic group, with a similar degree of restoration. However, the ADSC + EGCG group showed better protective effects with nearly all empty spaces filled. Quantitative analysis also confirmed that ADSC group showed decreased tissue disorder, whereas ADSC + EGCG group showed significantly decreased tissue disorder in the cortex (**Figure 2B**). Interestingly, results of ADSC + mimic and ADSC + inhibitor groups were very similarly to that of the ADSC group. These findings suggested that while ADSCs provided some protective effects, ADSC + EGCG group showed more beneficial effects in the aging rat cortex.

### EGCG-preconditioned ADSCs up-regulate the expression of survival-related proteins and proteins involved in molecular pathways for longevity in aging rat cortex

**Figure 3:**
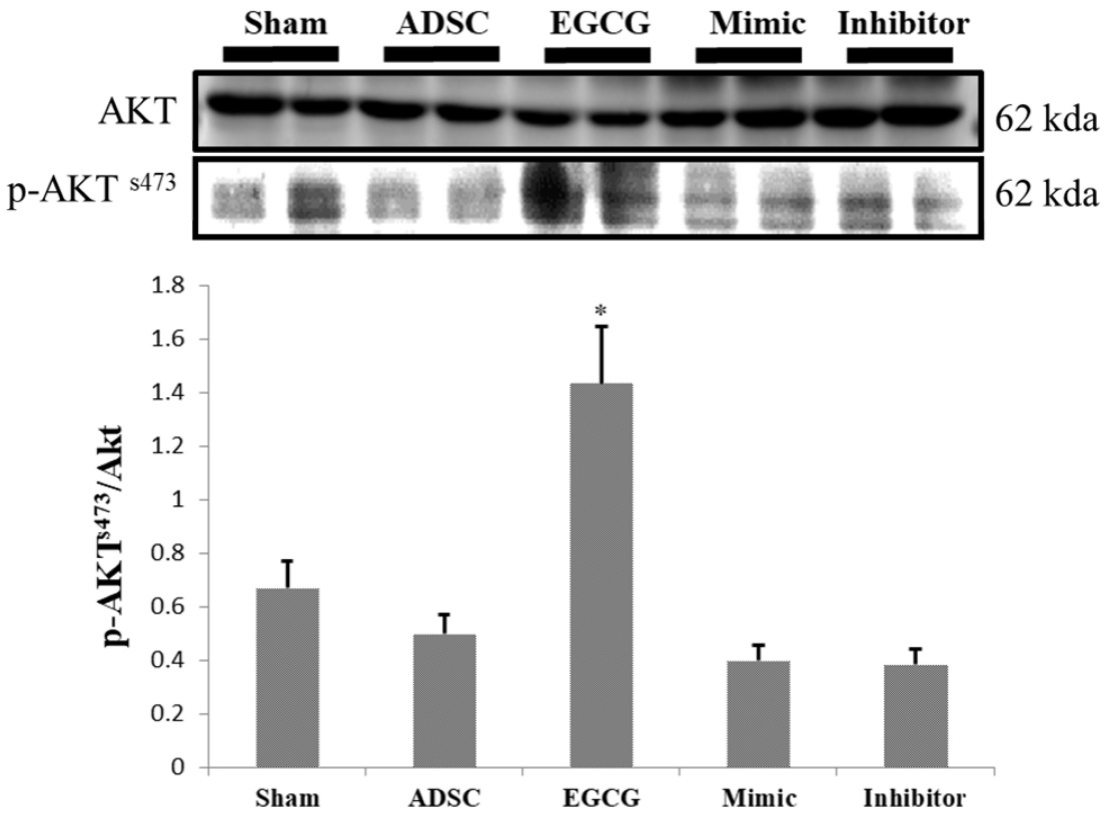
ADSC + EGCG group was able to up-regulate p-AKT to the highest degree, with the ADSC + mimic and ADSC groups at a close second. Data are presented as the mean ± SEM of three independent tests, and each group contained two rats. **p < 0.01 vs control group.

**Figure 4:**
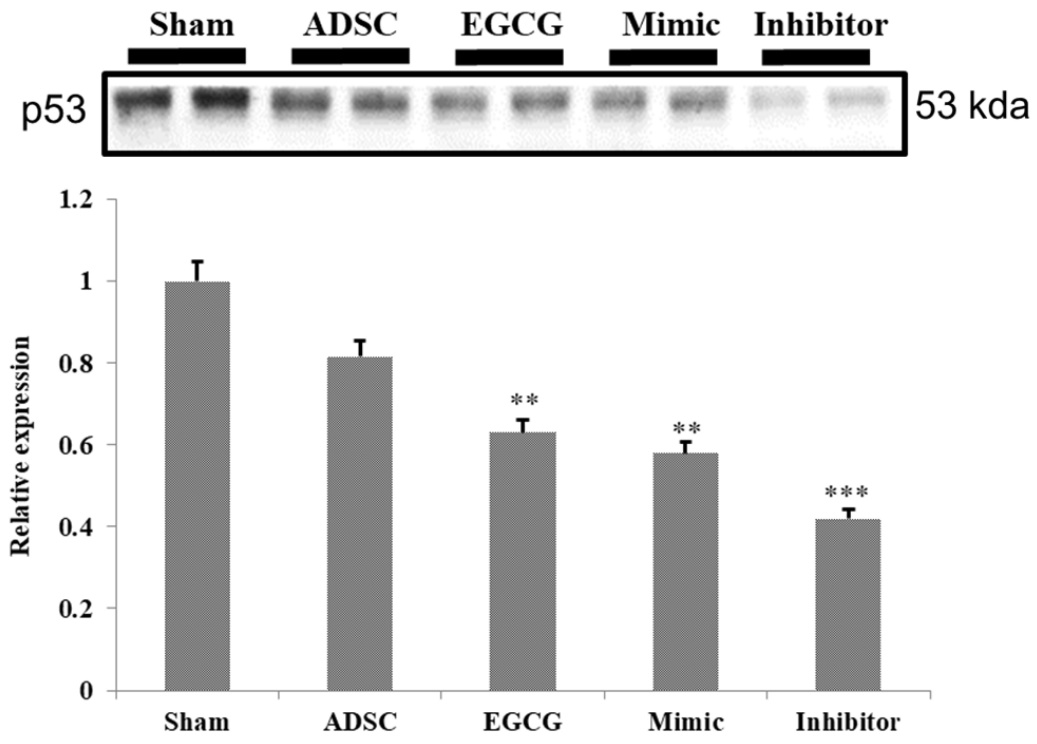
Quantification data revealed that the levels of the cell cycle regulator and tumor suppressor, P53, decreased in all experimental groups. Data are presented as the mean ± SEM of three independent tests, and each group contained two rats. ***p < 0.001 vs control group.

**Figure 5:**
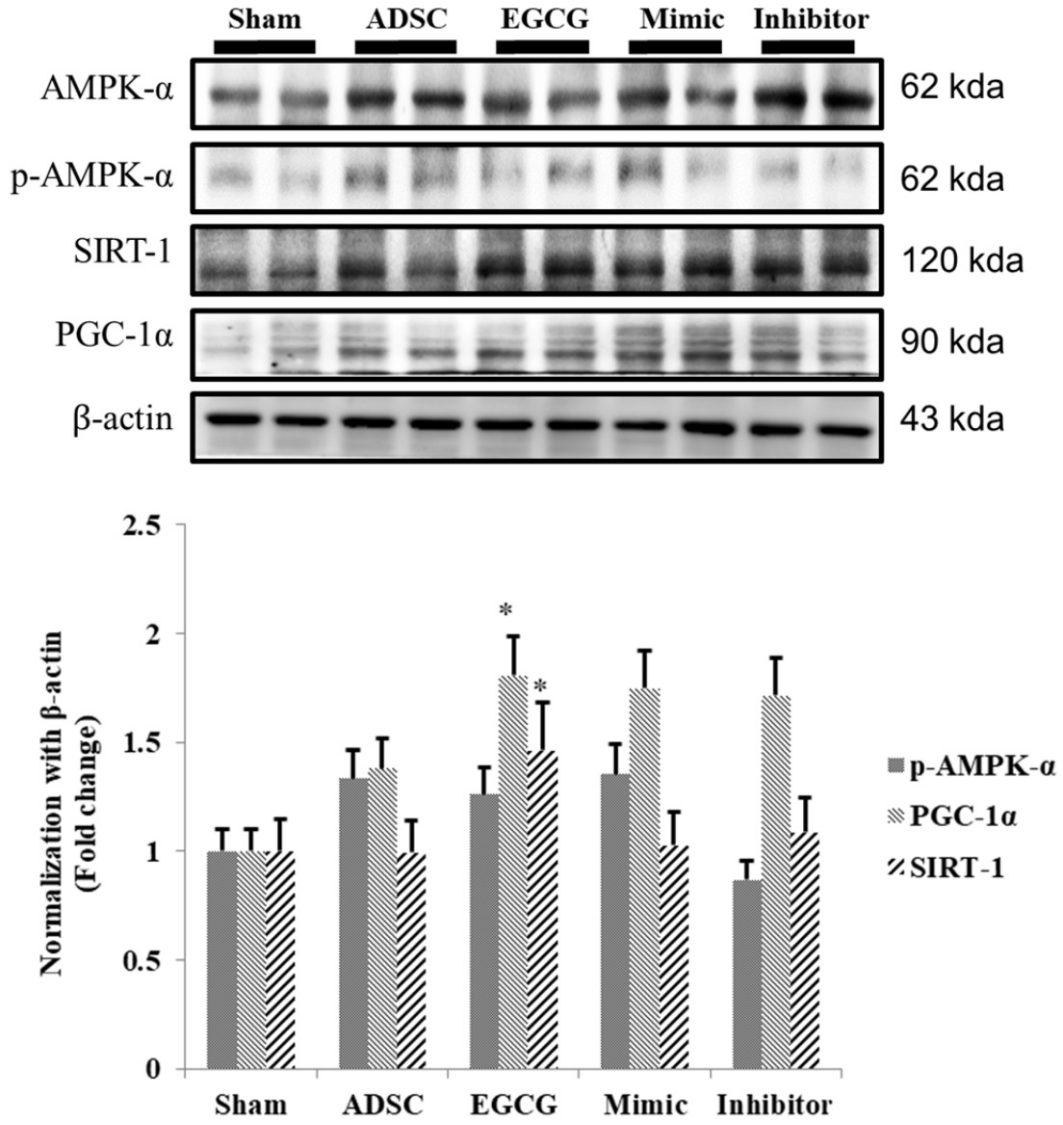
Quantitative analysis showed that SIRT-1, PGC-1α, and p-AMPKα tend to increase together, though at different rates. ADSC + EGCG group exhibited the highest up-regulation of these three proteins. Data are presented as the mean ± SEM of three independent tests, and each group contained two rats. *p < 0.05, **p < 0.01 vs control group.

To identify the molecular mechanism underlying the effects of ADSCs and EGCG, we analyzed the expression of the relevant proteins. We found no significant change in the expression of Akt between the groups (**Figure 2**). Importantly, phosphorylated Akt (p-Akt), a survival-related protein, was significantly increased in ADSC + EGCG group compared to that in other groups (**Figure 3**). Moreover, the ratio of p-Akt/Akt was significantly higher than that in ADSC group. Next, we examined the expression of P53, an apoptosis-related protein, and found it to be higher in the control group as compared to that in other treatment groups (**Figure 4**). Moreover, the expression of p-AMPKα, SIRT-1, and PGC-1α was increased in ADSC and ADSC + EGCG groups compared to that in the control group (**Figure 5**). Importantly, expression of these proteins involved in molecular pathways that promote longevity was higher in ADSC + EGCG group than that in ADSC group. These finding suggested that EGCG improved the beneficial effects of ADSC in protection against aging injury by enhancing the expression of p-Akt and proteins involved in molecular pathways promoting longevity in rat cortex, but no effect was observed in ADSCs preconditioned with mimic and inhibitor.

### EGCG-preconditioned ADSCs increased anti-oxidant molecular mechanism in aging rat cortex

**Figure 6:**
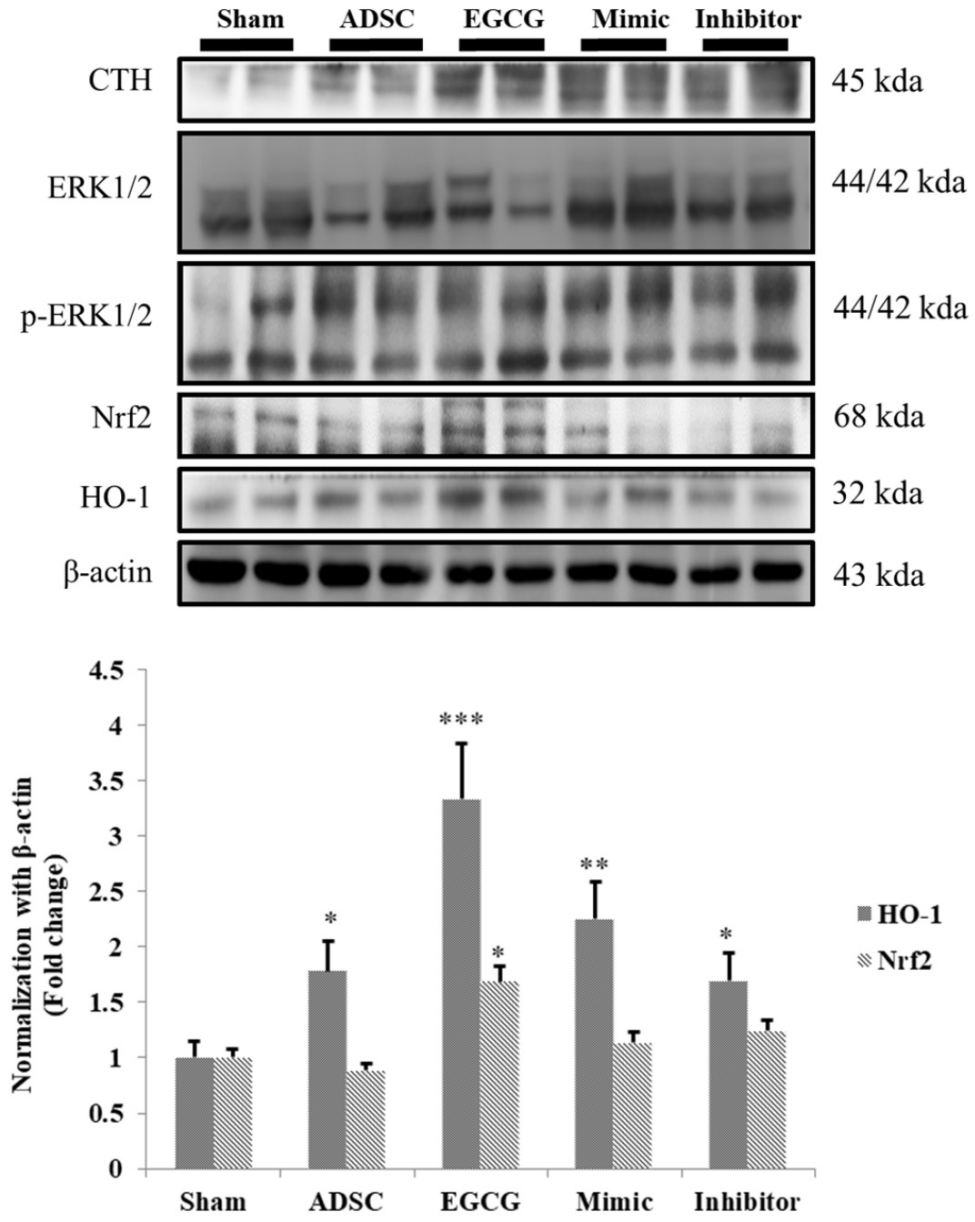
Nrf-2 & HO-1 quantification revealed that ADSC + EGCG group showed the highest up-regulating effect on Nrf-2 and HO-1 expression, with a minor decrease seen in the ADSC group. Data are presented as the mean ± SEM of three independent tests, and each group contained two rats. *p < 0.05 vs control group.

ROS accumulation is an important issue in aging brain, and therefore we examined the expression of cystathionine gamma-lyase (CTH), a glutathione pathway related protein, and anti-oxidant related proteins HO-1 and Nrf-2. We found that the expression of CTH was increased in ADSC and ADSC + EGCG groups, while that of HO-1 and Nrf-2 was up-regulated in the ADSC + EGCG group (**Figure 6**). These results indicated that EGCG-preconditioned ADSCs prevented aging injury in rat cortex by enhancing anti-oxidant related molecular factors.

### EGCG enhanced ADSC capacity to activate BDNF signaling pathway in aging rat cortex

**Figure 7:**
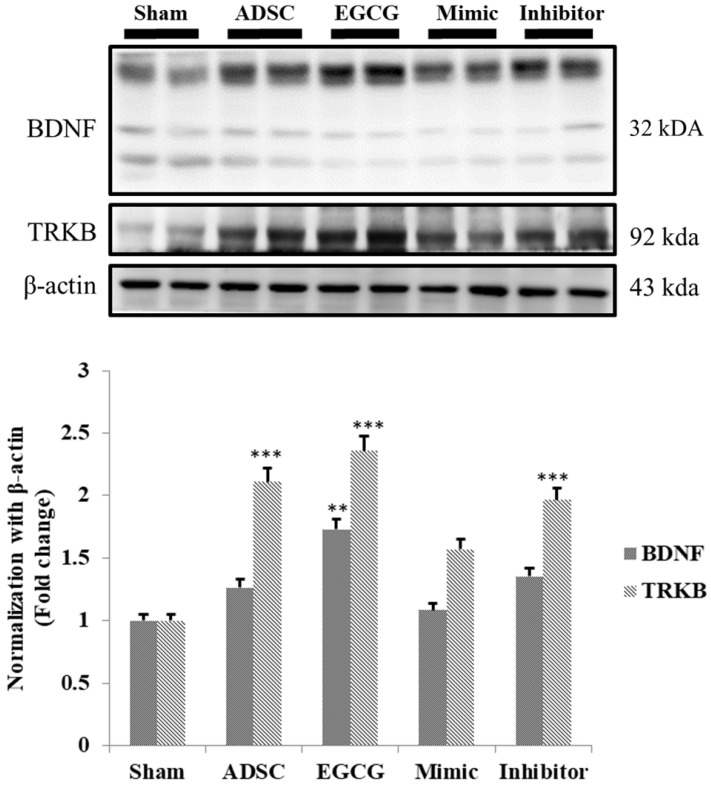
Quantification data showed a strong positive correlation between the increased TrkB and the increase in serum levels of BDNF precursor, Pro-BDNF, and an inverse correlation with the active form of BDNF. Data are presented as the mean ± SEM of three independent tests, and each group contained two rats. ***p < 0.001 vs control group.

As the results indicated that expression of pro-survival and anti-oxidant proteins in aging rat cortex in ADSC + EGCG group was higher than that in control group and better than that in ADSC group, we further examined whether ADSCs and EGCG-preconditioned ADSCs could improve the neurotrophic molecular pathway in aging rat cortex. Expression of neurotrophic related proteins such as BDNF and tyrosine kinase B (TrkB) was up-regulated in ADSC and ADSC + EGCG groups compared to that in control group (**Figure 7**). Moreover, expression of BDNF and TrkB in ADSC + EGCG group was higher than that in ADSC group. These findings suggested that EGCG-preconditioned ADSCs provided more beneficial effects in aging cortex by activating neurotrophic molecular pathway.

### Recognized miRNA-3575 targets exhibited no change as observed by western blotting

**Figure 8:**
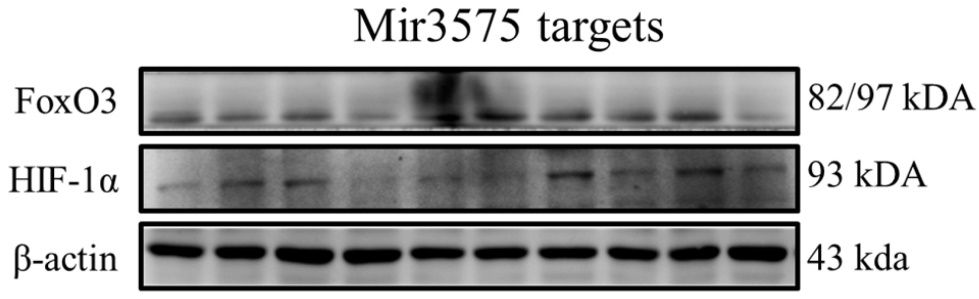
No substantial change was detected in the expression of miRNA targets, Hif-1 and FoxO3a. The signaling pathways involved may have been obscured or the target cells used in this experiment may not be suitable for miRNA-3575. It is also likely that, that as the experimental conditions were not hypoxic, Hif-1 was not induced and its effect was thus not revealed.

To determine whether the beneficial effect of EGCG was mediated via regulation of microRNA expression, we analyzed the expression of miRNA-3575 targets Hif-1α and FoxO3a. Our results showed that their expression was not changed (**Figure 8**). In addition, similar to the results of H&E staining, we found that ADSCs preconditioned with miR-3575 mimic or inhibitor did not provide more beneficial effects in aging rat cortex. Taken together, our findings suggested that EGCG could enhance ADSC activity to decrease aging-related injury in rat cortex, but not through microRNA-related mechanisms.

## Discussion

EGCG is an important and promising polyphenol involved in mechanisms that can positively affect the entire body. However, its intake through consumption of green tea rarely provides high enough doses to cause a strong neural effect, as the concentration of EGCG that crosses the blood brain barrier is only about 2.8% of the total consumed quantity in 30 min 14. In this study, we identified novel method for introducing this catechin into an aged rat model by co-culturing young ADSCs, obtained from the animal, with EGCG and subsequently re-introducing these preconditioned cells into them. The results showed that the most beneficial effect in aged rat cortex was observed when ADSCs and EGCG were combined and injected intravenously. It is possible that the ADSCs were able to permeate the blood brain barrier more effectively than EGCG alone, and thus, once inside the cortex, the factors from the ADSCs preconditioned with EGCG may have been secreted out as the ADSCs proliferated. In addition, EGCG potentially increased the robustness and proliferation of ADSCs by increasing cell proliferation signaling and mitochondrial biogenesis.

In the present study, the ADSC + EGCG group showed a marked improvement in the integrity of the aged cortex tissue, whereas the control was plagued with lesions of empty space indicative of shrinkage in the brain. This may be attributed to EGCG creating an environment of increased neuron survival and inhibiting Nogo-A, a powerful myelin-derived neurite outgrowth inhibitor 28. BDNF and TrkB, a BDNF receptor, were found to be upregulated and may have contributed to this supportive environment for neuronal growth. Moreover, active BDNF levels decreased sharply, indicating an uptake of this nerve growth factor by the budding cells. At the same time, the ADSCs not only helped repair the brain tissue, but also triggered the neighboring neurons to do so. Similar findings have been reported where stem cell-secreted factors were shown to have a rejuvenating effect on tissues 12.

Further, proteins involved in the molecular pathways of longevity, SIRT-1, PGC-1α, and p-AMPKα, were significantly up-regulated in ADSC + EGCG group. These proteins have been implicated in improving and regulating mitochondrial function and turnover via increasing the Nuclear Respiratory Factor, which helps to regulate mitochondrial transcription. The effects of SIRT-1 are still debatable, in part because its effects are cell-dependent; however, several studies have suggested the importance of these three proteins in extending the mammalian lifespan, as they are also induced by other life-preserving compounds and practices, such as consumption of resveratrol, which increases AMPK levels, and dietary restrictions, which up-regulate SIRT-1 29. One reason could be that SIRT-1 is known to deacetylase p53, and their levels may be inversely correlated in the cortex tissue 19. However, p53 is also known to be up-regulated in the presence of increased ROS, as seen in the aging control group, and was strongly ameliorated in the ADSC + EGCG group; its levels may have dropped for this reason. Moreover, p-AKT^s473^ is a central protein that is targeted by the mTORC2 and PI3Kinase pathways and is integral in enhancing cell survival and angiogenesis via its up-regulation. The finding of the present study suggested that EGCG improved the beneficial effects of ADSC by enhancing the expression of p-Akt.

Although EGCG may promote ROS production, it also acts as an antioxidant and helps the body's natural defenses to protect the cells and DNA from ROS. This increase in ROS levels may explain the increased expression of Nrf-2 and HO-1 when EGCG preconditioned ADSCs were administered, but only a very slight decrease in both, as well as in p-ERK, was observed when ADSCs alone were administered. This could be explained by the possibility that the ADSCs indeed slightly alleviate the increased oxidative stress in the aged brain via their inherent antioxidant properties, allowing the Nrf-2 pathway to down-regulate somewhat, as previously reported 30. Because the Nrf-2 pathway may be activated through other means as well, EGCG administration was able to produce a detectable result. However, the decrease was not considered significant, as quantitative analysis revealed the results to be too close to those of the control group.

## Conclusion

In this study, we attempted to combine EGCG and ADSCs, both known to induce neurogenesis, to treat aging rats. We demonstrated that EGCG could influence ADSCs and enhanced their beneficial functions in aging rats by activating Akt, anti-oxidant pathway, and BDNF-related pathway in the cortex. These findings suggested that EGCG may be considered as a pretreatment for ADSCs before stem cell therapy in aging brain, which may facilitate functional recovery and enhance neuroprotection.
